# Bibliometric analysis of case-control studies on the association between HLA alleles and multiple sclerosis in adults

**DOI:** 10.3389/fgene.2025.1524360

**Published:** 2025-03-11

**Authors:** Enola Maer, Marjorie Maya Hubacher, Livia Livint Popa, Dana Marieta Fodor, Razvan Mircea Chereches, Dafin F. Muresanu, Vitalie Vacaras, Maria Chiriac, Horea Vladi Matei, Nicu Catalin Draghici, Adrian Florea

**Affiliations:** ^1^ Department of Clinical Neurosciences, Iuliu Hatieganu, University of Medicine and Pharmacy, Cluj-Napoca, Romania; ^2^ RoNeuro Institute for Neurological Research and Diagnostics, Cluj Napoca, Cluj, Romania; ^3^ Center for Molecular Medicine, Karolinska Institutet, Solna, Stockholm, Sweden; ^4^ Department of Public Health, Babeș-Bolyai University, Cluj-Napoca, Cluj, Romania; ^5^ County Emergency Hospital Cluj-Napoca, Cluj-Napoca, Cluj, Romania; ^6^ Department of Molecular Sciences, Iuliu Hațieganu University of Medicine and Pharmacy, Cluj-Napoca, Romania; ^7^ IMOGEN Institute, Centre of Advanced Research Studies, Emergency Clinical County Hospital Cluj, Cluj-Napoca, Romania

**Keywords:** HLA, multiple sclerosis, MS, bibliometric analysis, HLA-DRB1, genetic susceptibility

## Abstract

**Background:**

Multiple sclerosis (MS) is a complex autoimmune disease that affects the central nervous system (CNS) with genetic and environmental factors playing a significant role in its development and progression. One of the most important genetic factors associated with MS is the HLA gene complex. The relationship between HLA and MS has been the subject of numerous studies, but no bibliometric analysis of this research has been reported to date. Therefore, this study aimed to provide a comprehensive overview of the publication output, citation impact, collaboration patterns, and research topics related to HLA and MS.

**Methods:**

A bibliometric analysis of 488 studies published between 1988 and 2023 was conducted using RStudio, Tableau and VOSviewer software.

**Results:**

The results indicated an increasing trend in the number of publications and citations over time, with the highest productivity and impact coming from researchers in the United States, Italy and Sweden. The analysis also revealed collaboration networks among researchers and institutions, with the most common research topics being the association of HLA alleles with MS susceptibility, disease course, and treatment response. This study’s limitations stem from the inherent biases associated with bibliometric analysis, including database and coverage bias, citation bias, and biases related to accessibility and open access. Additionally, the exclusion of non-English language articles represents a further limitation.

**Conclusion:**

Overall, this bibliometric analysis provides valuable insight into the research landscape of HLA and MS, highlighting the areas that have received the most attention and identifying potential avenues for future research.

## 1 Introduction

Multiple sclerosis (MS) is a complex neurological disorder that affects millions of people worldwide.

It is a complex autoimmune disease that affects the central nervous system (CNS) and is characterized by inflammation, demyelination, and axonal damage. The etiology of MS is not fully understood, but it is thought to be a multifactorial disease, with genetic and environmental factors contributing to its development and progression. One of the most significant genetic factors associated with MS is the human leukocyte antigen (HLA) gene complex. In 1972, prior to the description of HLA class II antigens, the initial studies linking HLA to multiple sclerosis (MS) were independently reported by three distinct groups. HLA genes play a crucial role in the immune response by encoding for proteins that present foreign antigens to immune cells, thereby initiating an immune response. Given the critical role of HLA genes in MS, it is not surprising that there has been substantial research on this topic over the years (Qiu et al., 2013). Genes beyond the HLA system also play a role, though small, in determining susceptibility to MS, but the magnitude of these effects has been observed to differ among various populations (Qiu et al., 2013).

Bibliometric analysis represents a methodological framework for quantitatively evaluating scientific literature, facilitating the objective assessment of publication patterns, dissemination impact, and collaborative networks within a specific research domain (L et al., 2023). In the context of bibliometric analysis, citation analysis is pivotal in assessing the scholarly impact and quality of scientific work. It also facilitates tracing knowledge dissemination, discerns emergent research trends, and elucidates collaborative networks. Moreover, citation metrics are instrumental for institutional benchmarking and strategic research planning, serving as indicators of the progression and shifts in scientific inquiry.

In bibliometric analysis, examining co-authorship, keywords, and co-citation is vital. Co-authorship analysis reveals the patterns of collaboration and intellectual networks within the global research community, indicating multidisciplinary efforts and key contributors. Keyword analysis identifies a field’s central themes and concepts, offering insights into the focus areas and emerging trends. Co-citation analysis uncovers synergies between scholarly works, highlighting the foundational papers and how research domains are interconnected. Together, these metrics provide a comprehensive understanding of the scientific landscape, guiding future research directions and policy-making (Donthu et al., 2021).

In this analysis, we have examined the publication output, citation impact, collaboration patterns, and research topics related to MS and HLA, using bibliometric methods to provide a comprehensive overview of the research landscape in this area.

Bibliometric analysis involves the consolidation and visualization of the bibliometric and intellectual framework of a particular field. This methodological approach investigates the relationships and networks among different research components, such as authors, countries, institutions, and topics. Adequate bibliometric studies can provide a solid foundation for advancing a field by identifying key influencers and patterns of collaboration. Currently, as an emerging technique, it has been widely applied across various medical domains, including neurology (Wilson et al., 2021; Li et al., 2008), lately in the field of multiple sclerosis (Aykaç and Eliaçık, 2022; Caballero-Villarraso et al., 2021).

Most bibliometric analyses focus on a single database. However, our study took a comprehensive approach by encompassing research from four of the most extensive databases, thereby ensuring a broader perspective.

## 2 Methods

### 2.1 Sources of data and search strategy

In March 2023, we implemented a detailed search strategy to collect data from a range of established databases, including the Web of Science Core Collection, PubMed, Scopus, and Embase. Our search strategy received validation from colleagues within the doctoral school. We chose the period 1988–2023 to capture a sufficiently long window of modern MS genetic research, beginning from when molecular HLA typing techniques became more widely adopted. Reviews and meta-analyses were excluded to ensure that our dataset comprised only original research findings for bibliometric analysis. This approach aligns with established bibliometric guidelines, which recommend focusing on primary articles to obtain a clearer view of original contributions within a field.

### 2.2 Web of science core collection

In the Web of Science Core Collection, our search, using the terms “HLA” and “multiple sclerosis” in titles (TI) or as author keywords (AK), yielded 1,105 articles

We refined this only to include articles published in English between 1988 and 2023 with specific relevance to the adult human population. Our strategy excluded studies involving animals, children, cell cultures and mice. The final search query we used was: ((((((((TI=(“HLA” AND “MULTIPLE SCLEROSIS”)) OR AK=(“HLA” AND “MULTIPLE SCLEROSIS”)) NOT TS=(animals)) NOT TS=(children OR pediatric)) NOT TS=(culture cells)) NOT TS=(“mice”)) AND LA=(English)) AND DT=(Article)) AND PY=(1988–2023).

### 2.3 PubMed

For our bibliometric analysis in PubMed, we used a search strategy combining “HLA” and “multiple sclerosis” as MeSH terms or in the article titles, yielding 617 articles

Our search was refined to include only studies on the adult human population, published in English, excluding reviews and meta-analyses. We focused on articles published between 1988 and 2023. Exclusions were made for articles with terms related to children, pediatrics, animals, cell cultures, and mice in their titles or abstracts. The final query was ((((((((((“HLA” [MeSH Terms]) AND “MULTIPLE SCLEROSIS” [MeSH Terms]) OR (“HLA” [Title] AND “MULTIPLE SCLEROSIS” [Title])) NOT “child” [Title/Abstract]) NOT “pediatric” [Title/Abstract]) NOT “animals” [Title/Abstract]) NOT “culture cells” [Title/Abstract]) NOT “mice” [Title/Abstract]) AND “english” [Language]) NOT (review [Publication Type])) NOT (meta-analysis [Publication Type])) AND ((“1988” [Date - Publication]: “2023” [Date - Publication])).

### 2.4 Scopus

In Scopus, our bibliometric analysis involved a search for articles with “HLA” and “multiple sclerosis” either in the title (TITLE) or as keywords (KEY), resulting in 3,491 articles

The search was refined to include English language articles only, published as research articles, and about the adult human population. We excluded content related to animals, children, pediatrics, and mice, and limited our search to articles published from 1988 onwards. The structured query was: TITLE (“HLA” AND “MULTIPLE SCLEROSIS”) OR KEY (“HLA” AND “MULTIPLE SCLEROSIS”) AND NOT “animals” AND NOT “children” AND NOT “pediatric” AND NOT “mice” AND NOT (PUBYEAR <1988) AND (LIMIT-TO (DOCTYPE, “ar”)) AND (LIMIT-TO (LANGUAGE, “English”)).

### 2.5 Embase

In Embase, our bibliometric analysis involved searching for articles with “HLA” and “multiple sclerosis” in the title (ti) or as keywords (kw), resulting in 885 articles

We limited this search to English language articles published as research articles relevant to the human adult population. Our search strategy excluded articles that mentioned animals, children, pediatrics, cell cultures, and mice in their abstracts (ab), and we focused on articles published from 1988 to 2023. The final query used was (“hla”:ti AND “multiple sclerosis”:ti OR (“hla”/kw AND “multiple sclerosis”/kw)) AND [english]/lim AND [article]/lim NOT “animals”:ab NOT “children”:ab NOT “pediatric”:ab NOT “cell culture”:ab NOT “mice”:ab AND [1988–2023].

### 2.6 Data screening

**FIGURE 1:**
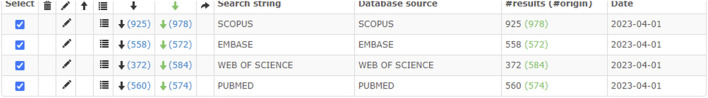
Cadima database

In the data screening phase of our bibliometric analysis, the refined search queries resulted in 574 articles from the Web of Science (WOS), 574 from PubMed, 978 from Scopus, and 572 from Embase as shown in Figure 1. Although our initial screening retrieved some studies mentioning specific MS subtypes (e.g., relapsing-remitting, primary progressive), the data were not uniform or sufficiently detailed to perform a reliable subgroup analysis by disease subtype. Similarly, although “female” and “male” appear as keywords, most publications did not provide detailed gender-stratified results suitable for quantitative synthesis. Hence, we did not conduct a separate gender-based bibliometric analysis in this study.

A complete list of the references analyzed in this study is provided in the supplementary materials accompanying this article.

### 2.7 Data analysis

**FIGURE 2:**
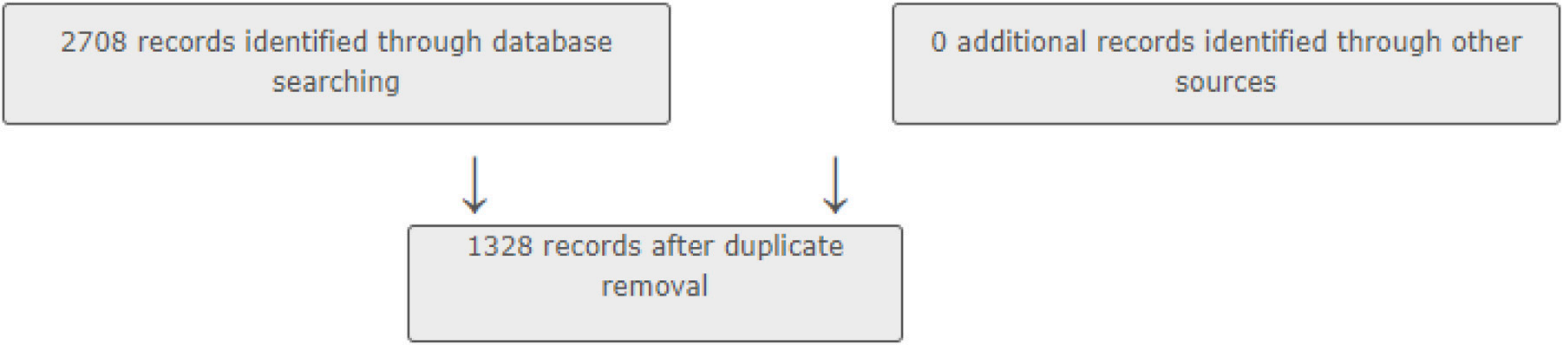
Flow diagram depicting the study selection process.

After running the query search in all four databases (n = 2,708), the overall data set was imported in CADIMA to remove the duplicates, ensuring that our analysis was based on unique entries. After removing them, all articles were uploaded to Mendeley for review (n = 1,328). Figure 2 provides a visual summary of the process described above.

Two reviewers systematically examined each article’s title and abstract, followed by a full-text review. Whenever there were discrepancies among the reviewers, a third reviewer was called upon to resolve these situations. This process identified 488 articles deemed eligible for analysis. The inclusion criteria focused on articles that studied and reported correlations between HLA complex alleles and multiple sclerosis in adults. Exclusion criteria were set to omit studies on animals, human cell cultures, reviews, and meta-analyses.

The 488 articles were included in our data analysis. RStudio provided a versatile means for statistical analysis and data processing. At the same time, Microsoft Office tools were utilized for organizing and managing the data, as well as preparing the final reports and presentations. Tableau and VOSviewer were used for their excellent capabilities in creating visual content, allowing us to present our data in an engaging and easily understandable format, through charts and graphics. This combination of tools facilitated an efficient and thorough analysis of our dataset.

Keywords were analyzed to identify popular topics in research on HLA haplotypes linked to MS. Keywords indicate articles research themes and co-occurring keywords reveal associations in the identified themes among the articles. The VOS method was applied to cluster keywords into different groups and each cluster was identified with a different color. Each word is represented by a circle, the diameter and label size denote the number of occurrences in titles, keywords or abstracts. Colors represent groups of linked terms, the label size of a term represents the number of publications on HLA haplotypes in which it is used, and the distance between two terms represents the degree to which they are associated.

Data were also analyzed in RStudio using libraries such as tidyverse, readxl, tidyr, dplyr, openxlsx, and ggplot2.

## 3 Results

The analysis yielded 488 records authored by 2,540 individuals, published across 127 journals, and encompassed 26,955 citations from references from 50 countries.

### 3.1 Global trends

**FIGURE 3:**
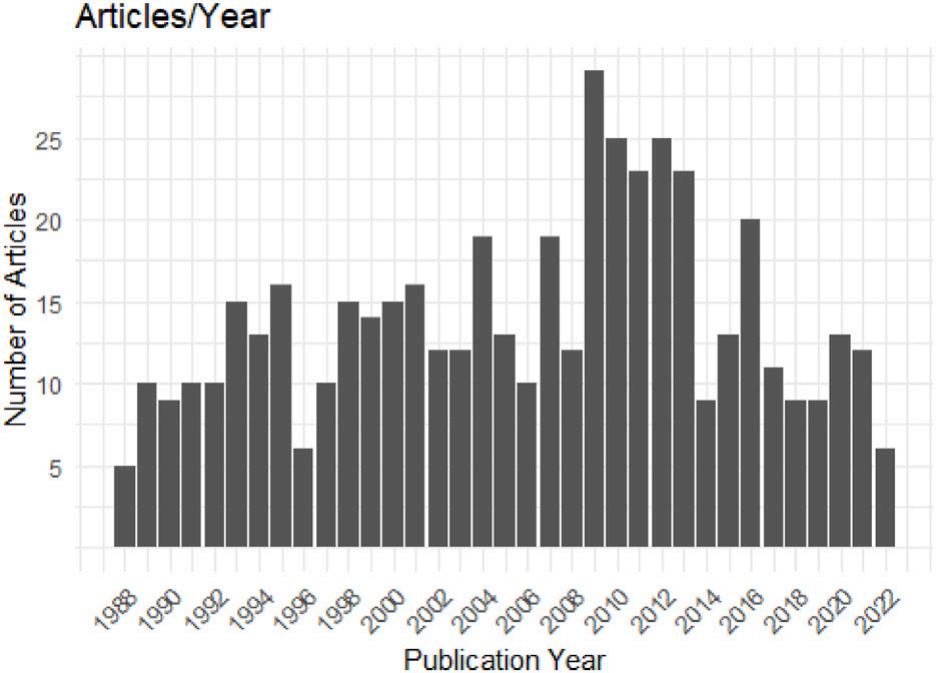
Global trends. Number of publications/year from 1988 to 2023.

Annually, the volume of research on this topic is increasing, with an annual average of 13.94 articles, a median of 13 articles, and a range of a minimum of 5 articles in 1988 to a peak of 29 articles in 2009. An exceptionally high concentration of publications was observed from 2009 to 2013. The average annual article count was 14.27 for 1998–2008, increased to 20.87 for 2009–2019, and then decreased to an average of 10 for 2017–2023 as seen in Figure 3. The average growth rate in publications between 1988–2023 stands at 9.87, which indicates the average annual increase in published studies throughout the evaluation.

**FIGURE 4:**
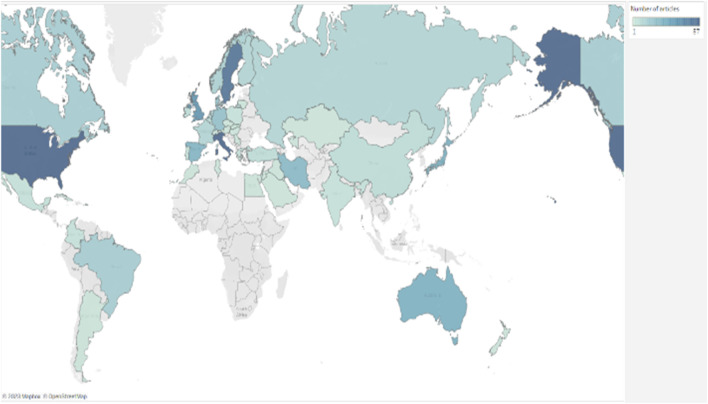
World map based on number of articles published in each country (darker = more articles). Source: Tableau.

The research encompassed studies from 50 countries, with Italy and the United States leading in publication volume, each contributing 11.7% of the total with 57 articles. Sweden followed closely with 10.2% and 50 articles. The UK also had a significant share of 7.79% with 38 articles, while Australia and Japan each accounted for 5.33% with 26 articles. Spain contributed 4.92% with 24 articles, Iran 4.71% with 23 articles, Germany 3.69% with 18 articles, and Denmark 3.48% with 17 articles. Figure 4 is a world map showing the distribution of published articles worldwide.

### 3.2 Analysis of journals

**FIGURE 5:**
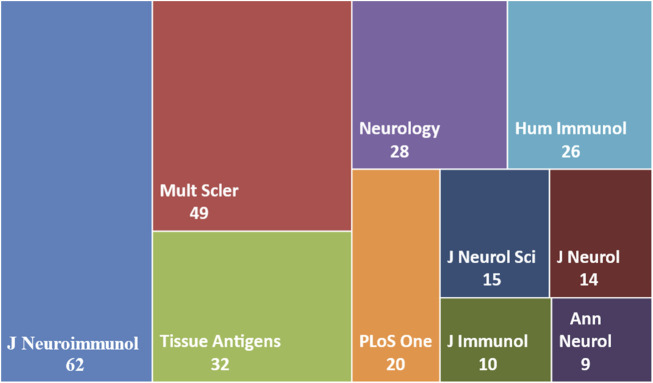
Analysis of the Journals. Top 10 Journals based on number of publications.

Original studies probing the connection between HLA haplotypes and Multiple Sclerosis have been published in 127 journals. The Journal of Neuroimmunology leads with 62 articles, Multiple Sclerosis Journal with 49 articles, and Tissue Antigens/HLA with 32 articles. Subsequently, Neurology published 28, Human Immunology had 26, and PLoS One featured 20 articles. The distribution of the top 10 journals based on the number of publications is illustrated in Figure 5.

Regarding citations, the Journal of Neuroimmunology ranks as the most cited with a total of 2,813 citations, averaging 45.37 citations per article. Neurology follows with 2,282 total citations and an average of 81.5 citations per article. The Multiple Sclerosis Journal has accumulated 2,143 citations with an average of 43.73 citations per article. Tissue Antigens/HLA has received 1,884 citations at an average of 58.87 citations per article, and Human Immunology stands at 1,417 citations with an average of 54.5 citations per article as presented in Table 1.

**TABLE 1:** Analysis of journals. Number of papers published. Total citations number. Impact factor.

Publication.Title	Nr of publications	Citations	Impact factor 2022	%
J Neuroimmunol	62	2,813	3,30	12,70
Mult Scler	49	2,143	5,80	10,04
Tissue Antigens/HLA	32	1884	4,51*	6,56
Neurology	28	2,282	9,90	5,74
Hum Immunol	26	1,417	2,21**	5,33
PLoS One	20	1,018	3,70	4,10
J Neurol Sci	15	788	4,40	3,07
J Neurol	14	591	6,00	2,87
J Immunol	10	1,251	4,40	2,05
Ann Neurol	9	1,390	11,20	1,84
*2021, **2021				

The symbols * and ** in Table 1 indicate that the impact factors for Tissue Antigens/HLA and Hum Immunol are from 2021 instead of 2022. This is because, at the time of our bibliometric analysis, the 2022 impact factors were not available in the sources we consulted.

### 3.3 Analysis and mapping of keywords

**FIGURE 6:**
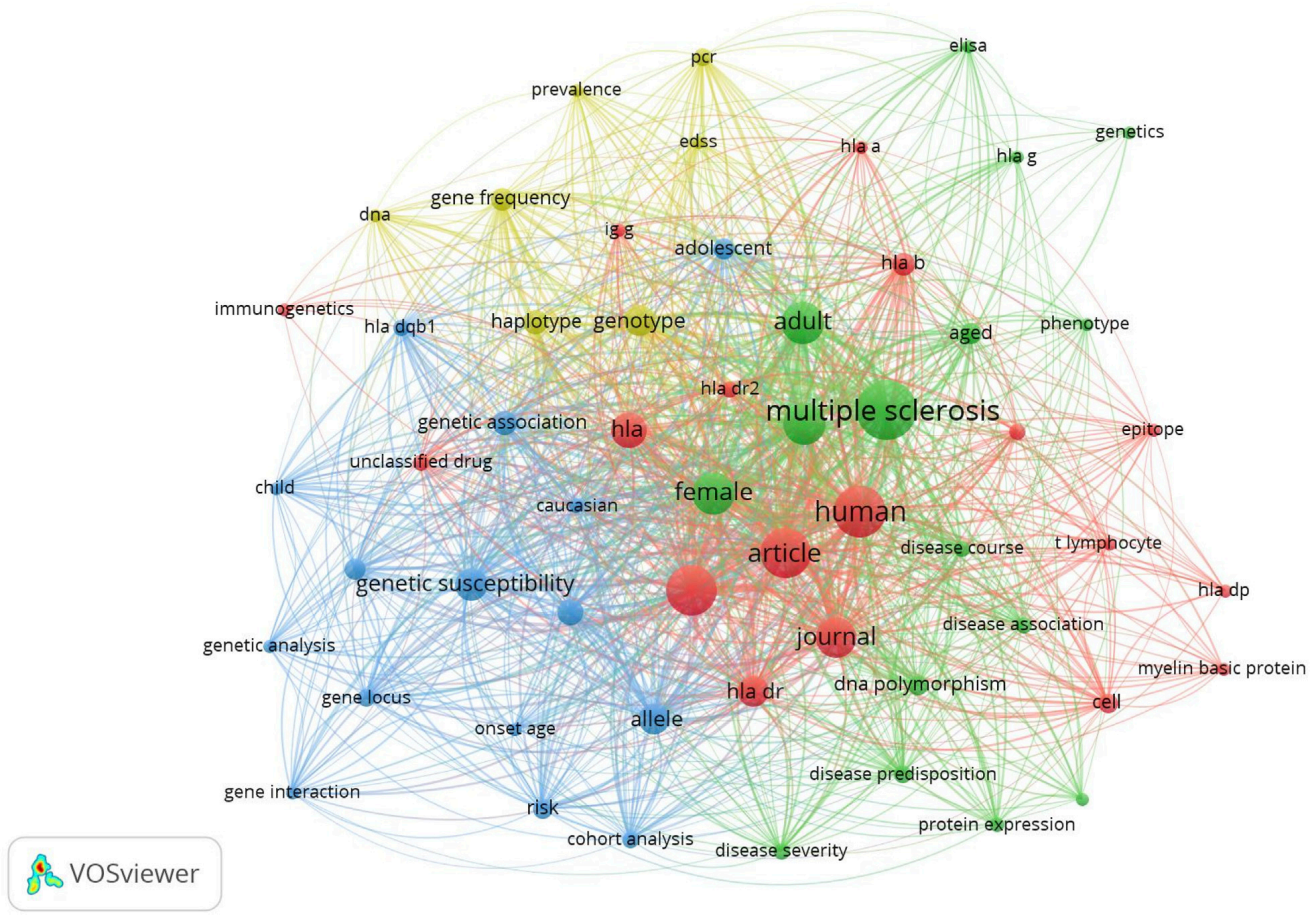
Analysis of Keywords. Mapping of keywords on studies on links between HLA haplotypes and MS.

The analysis of keywords from the 488 publications evaluated in this study was performed using VOSviewer software. Of the 762 keywords, 56 met the criterion of appearing more than five times across titles, abstracts, and keyword sections. These 56 keywords were organized into four clusters based on their occurrences, co-occurrences, links, and distinct items, resulting in 1,096 links and a total link strength of 6,859, as presented in Figure 6.

Cluster 1 (colored red) encapsulated 18 terms, notable among them were “human” with 81 occurrences and 55 links, “controlled study” with 75 occurrences and 54 links, “HLA” with 40 occurrences and 52 links, and “HLADR” with 31 occurrences and 50 links. Cluster 2 (green) compiled 16 terms, including “multiple sclerosis” with 112 occurrences and 55 links, “female” with 56 occurrences and 55 links, and “male” with 53 occurrences and 55 links.

Within Cluster 3 (blue), 15 terms were identified, among them “genetic susceptibility” with 32 occurrences and 50 links, “HLADRB1″ with 21 occurrences and 47 links, and “allele” with 30 occurrences and 52 links. Cluster 4 (yellow) assembled 7 terms, including “haplotype” with 19 occurrences and 49 links, “genotype” with 31 occurrences and 51 links, and “gene frequency” with 17 occurrences and 47 links.

**FIGURE 7:**
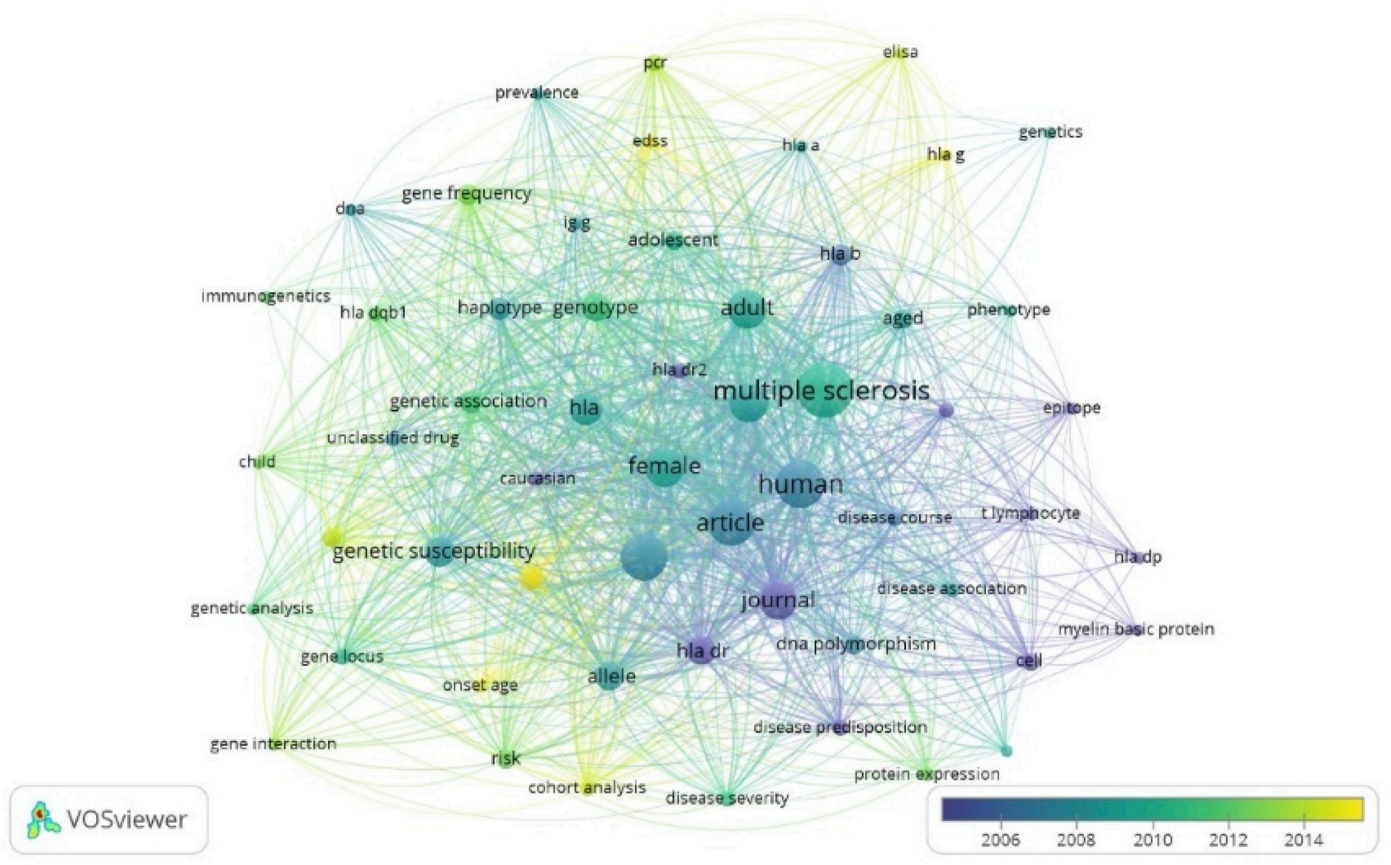
Analysis of Keywords. Distribution of keywords based on average publication year (purple earlier, yellow-recent). Source VosViewer.

The keywords were analyzed about their publication years, as illustrated in Figure 7. It represents a network visualization, revealing the central theme of MS and its strong association with genetic susceptibility and immunogenetics. The timeline of colors shows the evolution and shifting focus of research over the years from purple in the early years to yellow in the recent years.

### 3.4 Analysis of authors

There were 2,540 authors in the 488 articles studied in the bibliometric analysis.

Our bibliometric analysis did not distinguish between first and last authors but instead included all contributing authors in our dataset. We counted the number of times each author appeared in studies related to HLA and multiple sclerosis, creating a ranking based on overall contribution. This approach ensured that both senior researchers and early-career scientists were given equal recognition if they actively contributed to the field. By focusing on total authorship participation rather than hierarchical positioning, we aimed to provide a more inclusive perspective on influential researchers within this domain.

We acknowledge that analyzing the impact of first and last (senior) authors can be informative for understanding research leadership trends. However, to maintain inclusivity and focus on overall scientific contributions, we treated all co-authors equally in this bibliometric approach.

The authors with the most published studies were Hillert Jan (n = 42, 8.60%), followed by Olsson Tomas (n = 26, 5.33%) and Oksenberg Jorge R (n = 24, 4.9%). Alfredsson Lars (n = 18, 3.69%), Kockum Ingrid (n = 18, 3.69%) and Harbo, H F (n = 18, 3.69%) as presented in Table 2.

**TABLE 2:** Shows the Top 10 authors with the most published papers. *—number of citations for the papers analyzed.

Author	Nr. Of papers	Nr. Of citations*	Percentage %	Affiliation	Country
Hillert, J	42	3,951	8,6	Karolinska Institutet	Sweden
Olsson, T	26	2,717	5,3	Karolinska Institutet	Sweden
Oksenberg, J R	24	2,616	4,9	University of California	United States
Alfredsson, L	18	1,520	3,7	Karolinska Institutet	Sweden
Harbo, H F	18	1,469	3,7	Oslo University	Norway
Kockum, I	18	1,526	3,7	Karolinska Institutet	Sweden
Hauser, S L	17	2,196	3,5	University of California	United States
Kira, J-I	15	1,232	3,1	Kyushu University	Japan
Marrosu, M G	15	935	3,1	University of Cagliari	Italy
Compston, D A S	14	1,197	2,9	University of Cambridge	UK

In the bibliometric analysis of 488 articles, 2,540 authors were identified. Jan Hillert led the count of published studies with 42 publications (8.60%), followed by Tomas Olsson with 26 (5.33%) and Jorge R. Oksenberg with 24 (4.9%). Lars Alfredsson, Ingrid Kockum, and H.F. Harbo each contributed 18 publications, accounting for 3.69% each.

**FIGURE 8:**
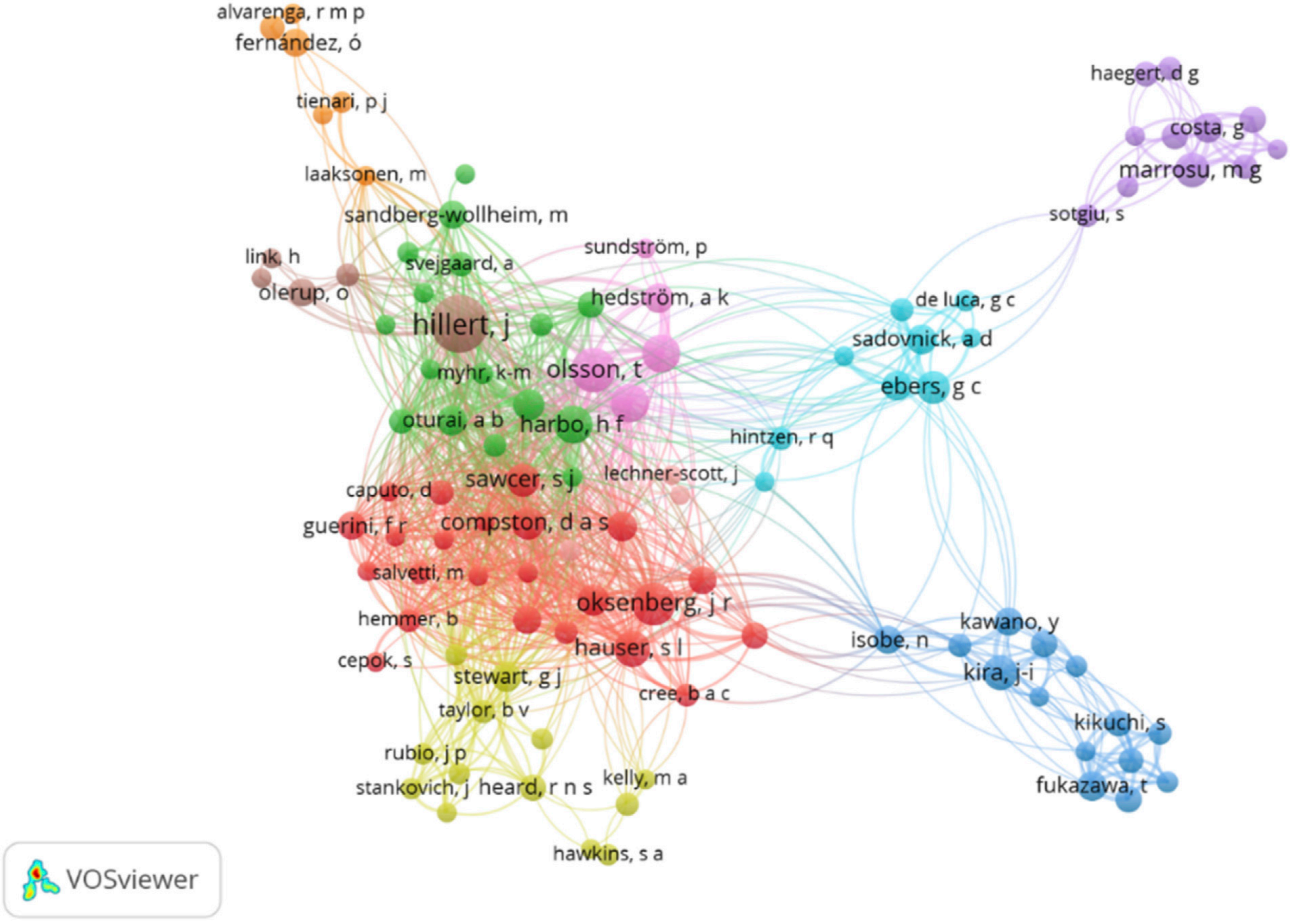
Analysis of authors based on co-authorship. Source: VosViewer.

Additionally, co-authorship patterns were examined using Vosviewer, setting the threshold at a minimum of 5 documents per author, with 121 out of the 2,540 authors meeting this criterion. The analysis revealed the total strength of co-authorship links, culminating in 105 connected items forming 10 clusters (1,058 links, total link strength: 2,573) as shown in Figure 8.

Cluster 1, represented in red by Vosviewer, included 23 items with notable figures such as J.R. Okensenberg (44 links, 144 total link strength), S.J. Sawcer (49 links, 130 total link strength), and D.A.S. Compston (47 links, 110 total link strength). Cluster 2, depicted in green, featured 16 items including H.F. Harbo (59 links, 174 total link strength) and, E.G., Celius (48 links, 131 total link strength). Cluster 3, in dark blue, showcased 14 items with N. Isobe (19 links, 50 total link strength) and J-I Kira (18 links, 57 total link strength).

In the yellow Cluster 4, 13 authors such as G.J. Stewart (41 links, 92 total link strength) and D. Booth (41 links, 82 total link strength) were highlighted. Cluster 5, in purple, gathered 11 items including S. Sotgiu (11 links, 21 total link strength) and M.G. Marrosu (10 links, 56 total link strength). The light blue Cluster 6 included 9 items with S.V. Ramagopalan (20 links, 50 total link strength) and G.C. Ebers (20 links, 55 total link strength). Cluster 7, in orange, featured 6 items with M. Laaksonen (19 links, 33 total link strength), and Cluster 8, in beige, had 5 items with J. Hillert standing out (61 links, 210 total link strength). Lastly, Cluster 9, in pink, included 5 items with T. Olsson (48 links, 163 total link strength), I. Kockum (45 links, 141 total link strength), and L. Alfredsson (38 links, 115 total link strength) among the noted authors.

### 3.5 Analysis of citations frequencies

Utilizing R Studio for citation analysis across different countries, it was found that the most cited research papers originated from the United States, with a total of 5,477 citations and an average of 96 citations per article. Sweden followed with 4,065 citations at an average of 81 per article, the UK with 2,587 citations (average of 68), Italy with 2,562 citations (average of 45 per article), and Japan with 1,788 citations (average of 68 per article) as presented in Table 3. The paper with the highest citation count is “A Complete Genomic Screen for Multiple Sclerosis Underscores a Role for the Major Histocompatibility Complex” by Haines J.L. et al., published in 1996, with 813 citations. This study, along with other highly cited papers, has significantly influenced the field by providing foundational insights into the genetic susceptibility of MS. The high citation count highlights its methodological robustness and its impact on subsequent studies exploring HLA-related MS susceptibility. This study established HLA-DR2 (now HLA-DRB1*15:01) as the most strongly associated genetic factor in MS. It pioneered genome-wide screens for MS susceptibility genes, influencing all subsequent genetic studies in the field. The second most cited study is “Class II HLA Interactions Modulate Genetic Risk for Multiple Sclerosis” (2015), a large-scale analysis of over 17,000 MS cases and 30,000 controls, which revealed key epistatic (interaction-based) effects between HLA risk alleles, demonstrating that their impact is non-additive. The third most cited study, “HLA-DR2 Dose Effect on Susceptibility to Multiple Sclerosis and Influence on Disease Course” (Barcellos et al., 2003), found that individuals with two copies of HLA-DRB1*15:01 had a significantly higher MS risk and worse disease progression. The fourth most cited study, “Mapping Multiple Sclerosis Susceptibility to the HLA-DR Locus in African Americans” (Hollenbach and Oksenberg, 2015), was one of the first to examine MS genetics outside European populations, demonstrating that HLA-DRB1 (not DQB1) is the primary risk gene in African Americans. The fifth most cited study, “Western Versus Asian Types of Multiple Sclerosis” (Kira et al., 1996), demonstrated that Western-type MS (brain involvement) and Asian-type MS (optic-spinal involvement) have different genetic associations, suggesting that MS is a heterogeneous disease across populations.

**TABLE 3:** Most productive 15 countries based on number of citations on papers that studies HLA-MS.

	Country	Nr of publications	%	Total citations
1	United States	57	11,68	5,477
2	Sweden	50	10,25	4,065
3	UK	38	7,79	2,587
4	Italy	57	11,68	2,562
5	Japan	26	5,33	1788
6	Germany	18	3,69	1,104
7	Australia	26	5,33	988
8	Norway	11	2,25	957
9	Canada	13	2,66	936
10	Spain	24	4,92	828
11	Denmark	17	3,48	706
12	Netherlands	9	1,84	659
13	Finland	10	2,05	489
14	Iran	23	4,71	472
15	France	10	2,05	457

**FIGURE 9:**
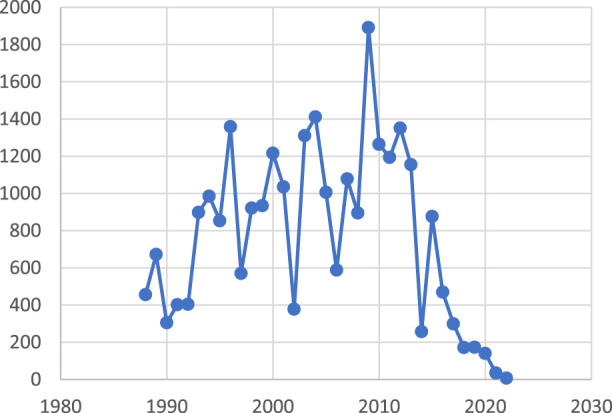
Distribution of citations over time (1988–2023)

Analysis revealed that the period from 2000 to 2012 saw the highest number of citations, indicating a concentrated period of significant research impact, as depicted in Figure 9.

### 3.6 Analysis of affiliations

A total of 227 institutions have contributed to the research on HLA and multiple sclerosis (MS), collectively producing 488 publications and accumulating 26,955 citations up to date. The analysis identified a highly concentrated research effort within a small subset of institutions, with the top 10 institutions accounting for 138 publications (28.3%) and 12,821 citations (47.5%). This suggests that a limited number of research centers play a disproportionately large role.

The Karolinska Institute emerged as the leading institution in terms of both publication output and citation impact, underscoring its pivotal role in HLA-MS research. Other key institutions, including the University of Oxford, Harvard University, and the University of California San Francisco, also demonstrated high research productivity and impact. Notably, while some institutions exhibited high publication volumes but lower citation rates, suggesting a focus on research output rather than scholarly influence, others, such as the University of California San Francisco, maintained a high citation-to-publication ratio, indicating a greater per-paper impact.

The geographic distribution of these high-impact institutions indicates a strong concentration in Europe and North America, with Sweden, the United Kingdom, and the United States hosting the most prolific research centers. The dominance of well-funded and internationally recognized institutions suggests that access to advanced genomic technologies, interdisciplinary collaborations, and robust funding mechanisms significantly contribute to research output and impact in the HLA-MS field.

**FIGURE 10:**
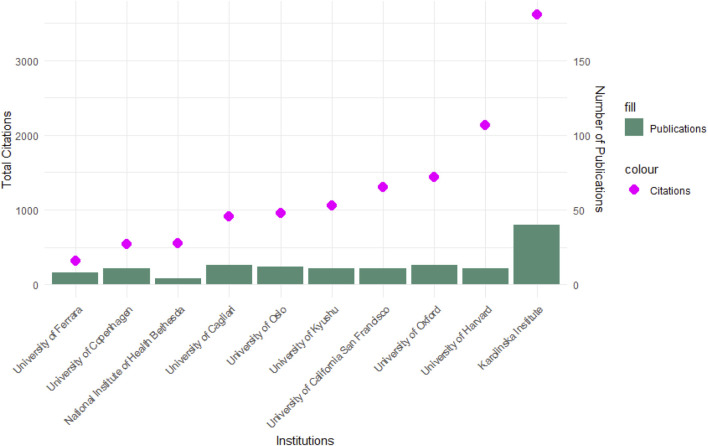
Top 10 institutions ranked by publication number.

Figure 10 presents the institutions ranked by the number of publications, highlighting their relative contributions to the field. The visualization illustrates the divergence between publication volume and citation impact, providing insight into the institutions shaping the global research landscape in HLA-MS.

## 4 Discussions

This study aimed to analyze trends in HLA haplotypes and multiple sclerosis research between 1988 and 2023. We reviewed four significant databases: Scopus, Web of Science, PubMed, and Embase.

Childhood MS was excluded due to limited data availability. Our bibliometric search across four databases yielded too few studies for robust analysis. Additionally, our study focuses on well-established HLA associations in adult MS, where more literature exists. Our findings revealed a publication growth, peaking between 1990–1995 and again between 2009–2016, before experiencing a recent downturn. The reasons for this decline remain uncertain, with possible factors including the COVID-19 pandemic or a decreasing interest in the subject area. Notably, 2009 was the peak year for publications and citations, with 29 articles receiving 1,891 citations.

The number of publications fluctuates over the years and exhibits variation across different countries. The United States, Sweden, Italy, and Japan are among the most prolific countries, contributing to 47% of the total publications This high-income countries dominate, largely due to well-funded research programs, international collaborations, and access to advanced sequencing technologies. Sweden has one of the highest incidences and prevalence rates of MS, which likely drives its strong research focus. Similarly, the United States also has a high MS incidence and prevalence, contributing to its leadership in MS genetics research. Italy, with its dense population and strong focus on neurology research, has made significant contributions, particularly in HLA-MS studies. Japan, despite having a lower prevalence of MS, has made substantial research contributions, particularly in studying Asian-specific MS subtypes and the role of genetic and environmental factors in disease susceptibility. The UK has been a leader in MS epidemiological research, with strong genetic studies and contributions to global MS databases and biobanks, further advancing HLA-MS research.

These findings reflect ongoing geographical disparities in MS genetics research, where high-income countries with robust funding and advanced research infrastructures (e.g., United States, Sweden, Italy) dominate publication and citation counts. Limited representation from low- and middle-income countries may stem from funding constraints, limited access to advanced genomic technologies, and fewer collaborative networks. Targeted capacity-building initiatives and international collaborations are vital to bridge this gap and enhance the global understanding of MS genetics.

Additionally, citation biases may arise due to the preference for English-language journals, self-citation practices, and the predominance of Western institutions in high-impact publications. These biases may inadvertently marginalize valuable research from non-English speaking or resource-limited settings.

Results of keywords co-occurrence analysis revealed that keywords were divided into four clusters. The one that exhibited high centrality were “multiple sclerosis,” “HLA,” “HLA-DRB1,” “HLA-DR,” “genotype,” “haplotype,” “genetic susceptibility,” “human,” and “DNA polymorphism,” serve as pivotal nodes that strengthen the framework of the research network within this scientific domain. Furthermore, we analyzed the development+ of keywords over time to reveal the frontiers and hotspots in the genetics of MS research. The results showed that the keywords such as “HLA G”, “EDSS” “gene interaction” “gene frequency,” “cohort analysis,” “protein expression,” and “HLA DQB1” began to appear in the previous decade significantly.

The journal with the highest number of publications and citations was the Journal of Neuroimmunology. Operating within the domain of clinical neurology, it is recognized as a Q1-tier journal, thanks to its recognizable academic reputation and global impact. This suggests that research in HLA genetics related to multiple sclerosis is of notable scientific value and quality. Furthermore, publishing in journals with global visibility and high impact factors fosters scholarly collaboration and knowledge sharing among researchers from various geographical locations. 54% of the papers analyzed were concentrated in the top 10 journals by publication volume. These journals cover several key disciplines, including neurology, immunology, immunogenetics, neuroimmunology, and multiple sclerosis research.

Potential implications for future research directions.

Most of the selected studies corroborate the well-recognized link between the DRB115–DQB10602 haplotype and multiple sclerosis. Moreover, the findings suggest that there are haplotypes that may exert a protective influence. Although several studies have identified putative protective alleles (e.g., HLA-A02:01, HLA-B44:02, and HLA-C*05), there is ongoing debate about their exact role in MS susceptibility and disease modulation (Hollenbach and Oksenberg, 2015). Some research groups report conflicting findings, possibly due to differences in cohort size, ethnic background, or methodological variations. These inconsistencies underscore the need for larger, multiethnic studies to clarify the precise impact of these alleles on MS risk. We included all case-control studies examining HLA associations with MS in adults, regardless of whether the authors reported risk alleles or protective alleles. No additional exclusion criteria were applied to papers solely on the basis of allele classification, ensuring that divergent findings on protective alleles were represented Research on polygenic diseases has concentrated on genes that predispose individuals to disease rather than those offering protection. Nonetheless, protective alleles or haplotypes can offer valuable biological and epidemiological understanding of the function of a disease gene.

Beyond case-control association studies, genetic methodologies can explore various aspects of the disease. However, the research is constrained by the reliance on small cohorts typically sourced from the same well-examined populations, coupled with a lack of extensive multicenter studies. Consequently, numerous regions remain unexplored in this research domain, leading to findings that may not be statistically relevant for those populations.

Genome-Wide Association Studies (GWAS) have the potential to significantly advance the field of gene-related research in multiple sclerosis (MS). The Human Genome Project and technological progress have enabled the creation of genetic variation catalogs and their efficient analysis. This led to a shift towards genome-wide association studies (GWAS), which compare genetic variants between patients and healthy individuals to find disease associations. GWAS use strict statistical thresholds (P < 5 × 10^−8) to ensure significance, considering the vast number of genome-wide comparisons (Lin et al., 2015; Uffelmann et al., 2021).

Reflection on the limitations of the bibliometric analysis.

Our study involved certain constraints which warranted consideration when evaluating our findings. The analysis was limited to publications in English, but it encompasses all significant and seminal works in the field.

Additionally, although we selected two persons to screen the initially identified publications, we cannot entirely rule out selection bias. We applied strict, pre-defined screening criteria, resulting in the exclusion of numerous non-conforming articles. Despite these limitations, our study shed light on the prospective research trends and emerging topics within this field to a certain extent.

Other limitations that may be taken into account are: citation practices as they vary by field and over time, omission of other scholarly contributions as bibliometric analysis typically focuses on journal articles, sometimes overlooking books, book chapters, conference papers, reports, and other literary output, inaccuracies in databases due to incorrect author names (we found a large number), affiliations, or citation details can lead to inaccurate attribution and counting of citations. We strove to mitigate bias by using multiple major databases and employing strict inclusion/exclusion criteria. Nonetheless, we acknowledge that publication bias, language restrictions, and the predominance of English-language journals may influence the results.

## 5 Conclusion

This study offers a comprehensive summary of critical information, areas of intense activity and emerging trends within the field of HLA-related genetics research in MS. Despite considerable advances in the analysis of HLA haplotypes associated with MS, there has been a decline in interest lately.

Offering valuable insights into publication trends, citation impact, and collaborative networks within this field. Our findings highlight the evolving focus of research over the past decades, as well as the pivotal role of HLA-DRB1 in MS susceptibility and disease progression.

Beyond its academic contributions, bibliometric analysis serves as a crucial tool for shaping research strategies, funding allocation, and policymaking. By identifying influential studies, emerging topics, and underexplored areas, this method aids researchers in setting priorities for future investigations. Additionally, funding agencies can leverage bibliometric insights to allocate resources effectively, ensuring investment in high-impact research areas. Policymakers can also utilize this data to develop evidence-based strategies for advancing MS research and improving patient outcomes.

Despite its inherent limitations, this bibliometric study underscores the importance of sustained collaboration between researchers, institutions, and countries to further investigate the genetic mechanisms underlying MS. The integration of advanced methodologies, such as Genome-Wide Association Studies (GWAS) and artificial intelligence-driven analytics, may enhance our understanding of MS genetics and open new avenues for research.

Ultimately, this study highlights the critical role of bibliometric analysis not only in assessing past research but also in guiding the future trajectory of scientific inquiry, fostering innovation, and enhancing the global impact of MS research.

Moving forward, these findings underscore the importance of sustained, collaborative efforts across different geographic regions. By leveraging new technologies such as GWAS, advanced bioinformatics, and machine learning, researchers and funding bodies can refine our understanding of MS genetics, develop targeted therapeutic strategies, and ultimately improve patient outcomes. Policymakers could use these bibliometric insights to allocate resources effectively, fostering global collaborations and ensuring that underrepresented regions gain equitable access to research infrastructure.

## References

[B1] Aykaç S. Eliaçık S. (2022). What are the trends in the treatment of multiple sclerosis in recent studies? - a bibliometric analysis with global productivity during 1980-2021. Mult. Scler. Relat. Disord. 68, 104185. 10.1016/j.msard.2022.104185 36183445

[B2] Caballero-Villarraso J. Sawas J. Escribano B. M. Martín-Hersog F. A. Valverde-Martínez A. Túnez I. (2021). Gene and cell therapy and nanomedicine for the treatment of multiple sclerosis: bibliometric analysis and systematic review of clinical outcomes. Expert Rev. Neurother. 21 (4), 431–441. 10.1080/14737175.2021.1886926 33554666

[B3] Donthu N. Kumar S. Mukherjee D. Pandey N. Marc W. (2021). How to conduct a bibliometric analysis: an overview and guidelines. J. Bus. Res. 133 (March), 285–296. 10.1016/j.jbusres.2021.04.070

[B4] Hollenbach J. A. Oksenberg J. R. (2015). The immunogenetics of multiple sclerosis: a comprehensive review. J. Autoimmun. 64, 13–25. 10.1016/j.jaut.2015.06.010 26142251 PMC4687745

[B5] L M. K. George R. J. Anisha P. S. (2023). Bibliometric analysis for medical research. 45(3):277–282. 10.1177/02537176221103617 PMC10159556 37152388

[B11] Kira J. Kanai T. Nishimura Y. Yamasaki K. Matsushita S. Kawano Y. (1996). Western versus Asian types of multiple sclerosis: immunogenetically and clinically distinct disorders. Ann Neurol. 40 (4), 569–574. 10.1002/ana.410400405 8871575

[B6] Li T. Ho Y. S. Li C. Y. (2008). Bibliometric analysis on global Parkinson’s disease research trends during 1991–2006. Neurosci. Lett. 441 (3), 248–252. 10.1016/j.neulet.2008.06.044 18582532

[B7] Lin X. Deng F. Y. Lu X. Lei S. F. (2015). Susceptibility genes for multiple sclerosis identified in a gene-based genome-wide association study. J. Clin. Neurol. 11 (4), 311–318. 10.3988/jcn.2015.11.4.311 26320842 PMC4596110

[B8] Qiu W. Pham K. James I. Nolan D. Castley A. Christiansen F. T. (2013). The influence of non-HLA gene polymorphisms and interactions on disease risk in a Western Australian multiple sclerosis cohort. J. Neuroimmunol. 261 (1–2), 92–97. 10.1016/j.jneuroim.2013.04.022 23726763

[B9] Uffelmann E. Huang Q. Q. Munung N. S. de Vries J. Okada Y. Martin A. R. (2021). Genome-wide association studies. Nat. Rev. Methods Prim. 1 (1), 59. 10.1038/s43586-021-00056-9

[B10] Wilson M. Sampson M. Barrowman N. Doja A. (2021). Bibliometric analysis of neurology articles published in general medicine journals. JAMA Netw. Open 4 (4), e215840. 10.1001/jamanetworkopen.2021.5840 33856477 PMC8050738

